# Clinical and laboratory markers defining MIS-C and hyperinflammation in COVID-19: a cross-sectional study in a tertiary hospital

**DOI:** 10.1186/s42358-025-00447-6

**Published:** 2025-03-17

**Authors:** Ana Paula Radünz Vieira, Paulo Roberto Antonaccio Carvalho, Sandra Helena Machado, Taís Sica da Rocha

**Affiliations:** 1 Pediatric Rheumatology Division, Federal University of Rio Grande do Sul, Hospital de Clínicas de Porto Alegre, Ramiro Barcelos Street, 2350 - Santa Cecília, Porto Alegre City, Rio Grande do Sul 90035-903 Brazil; 2 Pediatric Intensive Care Division, Federal University of Rio Grande do Sul, Hospital de Clínicas de Porto Alegre, Ramiro Barcelos Street, 2350 - Santa Cecília, Porto Alegre City, Rio Grande do Sul 90035-903 Brazil

**Keywords:** MIS-C, Hyperinflammation, COVID, Children, BNP, B-type natriuretic peptide, Troponin

## Abstract

**Background:**

Numerous inflammatory complications related to COVID are described, including the Multisystem inflammatory Syndrome in Children (MIS-C) and Hyperinflammation. There is a scarcity of studies comparing these two groups.

**Methods:**

Retrospective longitudinal outcome-conditioned study. Demographic, clinical, and laboratory variables are analyzed. Patients with history of COVID contact or infection with at least 24 h of fever, two or more systems involved and up to 21 years were included. Patients with no laboratory signal of inflammation or with other diagnoses for the condition were excluded. Demographic and laboratory data are presented as medians with interquartile ranges. Dichotomous variables and prevalences are reported as percentages. A ROC curve analysis was conducted to assess the discriminatory ability of these tests in relation to the MIS-C and hyperinflammation groups.

**Results:**

We present fifty-four patients, thirty-one with MIS-C and twenty-three with hyperinflammation. The most frequent symptom in the MIS-C group was altered mental status in 61% vs. 46% (*p* = 0.014) and conjunctival hyperemia in 29% vs. 4% (*p* = 0.032). The most frequent laboratory findings were hypoalbuminemia in 68% vs. 26% (*p* = 0.002), increased serum troponin in 42% vs. 26% (*p* = 0.034), increased d-dimers in 94% vs. 76% (*p* = 0.015), as well as increased BNP in 55% vs. 17% (*p* = 0.02). On the other hand, the hyperinflammation group more frequently presented respiratory dysfunction in 57% vs. 13% (p = < 0.001) and serum ferritin equal or greater than 500 ng/mL in 94% vs. 77% (*p* = 0.046).

**Conclusions:**

This is an original study comparing clinical and laboratory findings between MIS-C and hyperinflammation due to COVID. Altered mental status is more frequently associated with MIS-C while respiratory symptoms are associated with hyperinflammation. In addition, regarding laboratory tests, there is hypoalbuminemia, increase in serum troponin, BNP, and D-dimers specially in the MIS-C group and hyperferritinemia in the hyperinflammation group. Further studies are needed to assess the cutoff point of biological markers such as BNP, troponin, and d-dimers for diagnosis and/or prognosis in the pediatric population with MIS-C.

**Supplementary Information:**

The online version contains supplementary material available at 10.1186/s42358-025-00447-6.

## Background

The pandemic caused by the coronavirus (since December 2019) initially presented reports of pneumonia rapidly evolving into severe acute respiratory syndrome in the city of Wuhan, China. The pathology soon escalated into a true global catastrophe with consequences that are still not fully understood [1]. In adult patients, the respiratory condition emerges as the primary manifestation, accompanied by multiple organ complications [2].

Adults with severe respiratory failure caused by SARS-CoV-2 infection typically experience clinical deterioration approximately one week after the initial exposure phase. At this stage, the virus enters the body by binding to angiotensin-converting enzyme 2 (ACE2) receptors present in blood vessels, lungs, intestines, and kidneys [2]. Adults exhibit a high expression of these receptors, resulting in increased viral production. In contrast, children have a lower expression of ACE2 receptors and, consequently, reduced viral replication [3].

The second stage of the infection (direct lung involvement) is less common in the pediatric population [3]. The inflammatory response is the third stage, presenting increased activation of macrophages, and stimulation by T-helper lymphocytes. Consequently, cytokines (such as interleukins, interferon, and tumor necrosis factor) are released, leading to further activation of macrophages, neutrophils, and monocytes (cellular innate response, the “immediate response”), as well as activation of plasma cells for antibody production (adaptive immune response, which takes longer to activate) [3]. This cascade results in a significant hyperimmune response, which is theorized to cause hyperinflammation (acute presentation) and Multisystem inflammatory Syndrome in Children (MIS-C) - late inflammatory presentation.

By mid-April 2020, a report of eight cases in the United Kingdom suggested that children with coronavirus infection might exhibit a significant immune activation either during the infectious process or approximately 4–8 weeks after exposure [4]. This inflammatory process was initially termed Multisystem Inflammatory Syndrome in Children (MIS-C) associated with coronavirus [3, 4]. Subsequently, in 2021, the concept of Acute COVID-19-Associated Hyperinflammation was introduced in the 2nd version of the American College of Rheumatology (ACR) clinical guideline for MIS-C [5].

Early reports of MIS-C describe similar characteristics to other known inflammatory syndromes: Kawasaki disease inflammatory syndrome, hemophagocytic syndrome, macrophage activation syndrome, and toxic shock syndrome [3, 6, 7].

Unlike Kawasaki disease, MIS-C appears to affect children older than 5 years and is more frequently associated with direct cardiac involvement, as myocarditis, rather than being restricted to blood vessels and coronary arteries as in Kawasaki disease [7]. Additionally, unlike hemophagocytic syndrome or macrophage activation syndrome, there are differences in the type of hematological involvement, with leukocytosis and thrombocytosis being more common in MIS-C cases [7].

Nakra et al. also reported that serological tests for COVID infection showed approximately 87% positivity, while RT-PCR positivity was about 32% in MIS-C cases, suggesting a delayed onset disease as compared to the primary COVID infection, supporting the hypothesis that the syndrome may be post-infectious rather than related to early acute infection [3, 6, 8].

In children, the clinical manifestations MIS-C and hyperinflammation (HI) tend to exhibit significant variability with a mortality rate ranging from 0.1 to 8% depending on the affected systems, population, level of development of the country, and patient’s unit of care — whether in the intensive care unit or a general pediatric ward [9, 10]. Fever is the cardinal symptom. Neurological findings such as headache, irritability, encephalopathy, as well as manifestations of peripheral neuropathy (e.g., Guillain-Barré syndrome), may be associated [11]. Mucosal involvement is also reported, including rash and aseptic conjunctivitis. Gastrointestinal symptoms are common and include vomiting, abdominal pain, and/or diarrhea. There have been reports of acute abdomen requiring exploratory laparotomy with findings of peritonitis and mesenteric lymphadenitis during the procedure [3]. Respiratory manifestations can range from the presence of rhinorrhea to respiratory failure requiring mechanical ventilation [3, 7, 11, 12].

Regarding cardiological involvement, echocardiograms demonstrate valve regurgitation, dilation of coronary arteries (and aneurysms), as well as reduced ejection fraction, myocardial involvement, and arrhythmias [13, 25]. Some children have developed hypotension, necessitating transfer to a pediatric intensive care unit and the use of vasoactive drugs [3, 7, 12]. Levels of troponin and B-type natriuretic peptide are usually elevated, indicating myocardial injury and heart failure, respectively [3, 7, 12]. Inflammatory markers such as C-reactive protein, erythrocyte sedimentation rate (ESR), procalcitonin, D-dimer, and/or ferritin are typically increased, along with elevated fibrinogen and decreased albumin in most cases, suggesting widespread inflammation [3, 7, 12]. Although thrombotic events are not directly described in the pediatric population, lymphopenia, and varying levels of platelets (low, normal, or elevated) have been observed [3, 7, 12].

Although the definition of MIS-C has been established since 2020 (with adaptations based on new developments in the field), it was not until 2021 that the American College of Rheumatology (ACR) introduced the concept of COVID-19-associated hyperinflammation: a state of immune activation during the acute phase of SARS-CoV-2 infection (also initially referred to as severe COVID-19) [5, 14]. This presentation is considered uncommon in the pediatric population but typically presents in patients with comorbidities such as malignancies or other severe chronic diseases [5]. Although there is no formal definition, hyperinflammation is always associated with a hyperactivated immune response known as a “cytokine storm,” characterized by varied clinical and laboratory parameters (including interleukins - primarily IL-1 and IL-6 - natural killer cells, CD4 and CD8 T lymphocytes, and even the purinergic metabolism of ATP release) [15].

It’s challenging to distinguish MIS-C and hyperinflammation due to overlapping of common features such as elevated inflammatory markers, fever, and constitutional symptoms (abdominal pain, vomiting, headache, etc.). However, they primarily differ based on the timing of these manifestations (more commonly observed during acute infection with positive viral tests in the case of hyperinflammation), the predominance of respiratory symptoms in hyperinflammation and the cytokine profile - though the access to the latter is difficult for most clinicians [5, 14, 15]. In addition to the previously described differences, a notable distinction is the response to treatment. Hyperinflammation typically shows little to no response to intravenous immunoglobulin (IVIG), unlike MIS-C, where IVIG is the first-line treatment [5, 14].

Although there are studies on MIS-C, there is still a lack of clinical data tailored to highlight and clarify the differences between MIS-C and hyperinflammation, as well as practical issues of identification that could help differentiate them from other inflammatory syndromes, thereby assisting in better clinical management.

## Methods

### Design study and patients

This study is a retrospective longitudinal cross-sectional study based on outcome through medical record analysis. MIS-C and Hyperinflammation were defined as CDC criteria from 2022 and ACR hyperinflammation definition and are described in the supplementary material [5, 14, 16].

The study was approved by the Ethics Committee of Research at the Federal University of Rio Grande do Sul. Patients up to 21 years of age treated at the Hospital de Clínicas de Porto Alegre with a history of contact or confirmed infection with SARS-CoV-2, with fever lasting more than 24 h and presenting symptoms in two or more organs or systems, during the period from March 2020 to December 2022 were included. Patients with other diagnosed conditions that could explain the symptoms or without laboratory evidence of inflammation were excluded.

The study population was identified through records of visits to the pediatric emergency department (for patients who did not require hospitalization), of admission to pediatric intensive care unit, and to pediatric inpatient unit, and flagged to the Pediatric Rheumatology team. After obtaining informed consent and assent when indicated, clinical and laboratory data were extracted from the review of the electronic medical records of patients.

### Definition

- **COVID-19-associated hyperinflammation** is defined by an increase in inflammatory markers, acute SARS-CoV-2 infection (up to 4 weeks), and involvement of two or more systems or organs [5, 14].
- **The CDC diagnostic criteria for MIS-C** used in 2022 are as described below [16]:

#### Clinical criteria

Individual under 21 years of age with:

- A minimum of 24 h of fever (objectively or subjectively) ≥ 38 °C AND.
- Severe illness or hospitalization required AND.
- 2 or more affected organs or systems (e.g., cardiac, renal, respiratory, hematological, gastrointestinal, dermatological, neurological).

#### Laboratory evidence of inflammation

One or more of the following: elevated C-reactive protein, erythrocyte sedimentation rate, fibrinogen, procalcitonin, D-dimer, ferritin, LDH, or interleukin-6; elevated neutrophils or reduced lymphocytes; low albumin.

#### Laboratory or epidemiological evidence of SARS-CoV-2 infection

- SARS-CoV-2 positive by real-time reverse transcription polymerase chain reaction (RT-PCR), serology, or antigens OR.
- Exposure to COVID-19 within the 4 weeks prior to the onset of symptoms.

#### Exclusion of other diagnoses

No alternative diagnoses.

Additional clinical and laboratory definitions are described in the supplementary material.

### Statistical analysis

Demographic data and laboratory values were presented as medians and interquartile ranges. Dichotomous variables and prevalences were reported as percentages. Data was processed and analyzed using R (R Core Team. R: A language and environment for statistical computing. R Foundation for Statistical Computing, Vienna, Austria, version 4.1.0), through the R Studio IDE (v 2022.2.2.485), and the SPSS statistical package (Statistical Package for the Social Sciences) version 18. Given that the objective of this study was to explore differences between the MIS-C and hyperinflammation (HI) groups regarding laboratory test results, differences between groups were calculated using the Mann-Whitney test, with adjustments for variance differences and, due to ties, the exact p-value was determined using the “coin” package. Qualitative variables were compared using Fisher’s exact test. In the assessment of laboratory tests, ROC curves were constructed to evaluate the discrimination of these tests between the MIS-C and hyperinflammation groups, with statistical significance considered at p-value < 0.05 and AUC-ROC > 0.5. The points of highest specificity and sensitivity were reported.

## Results

### Population

**Fig. 1:**
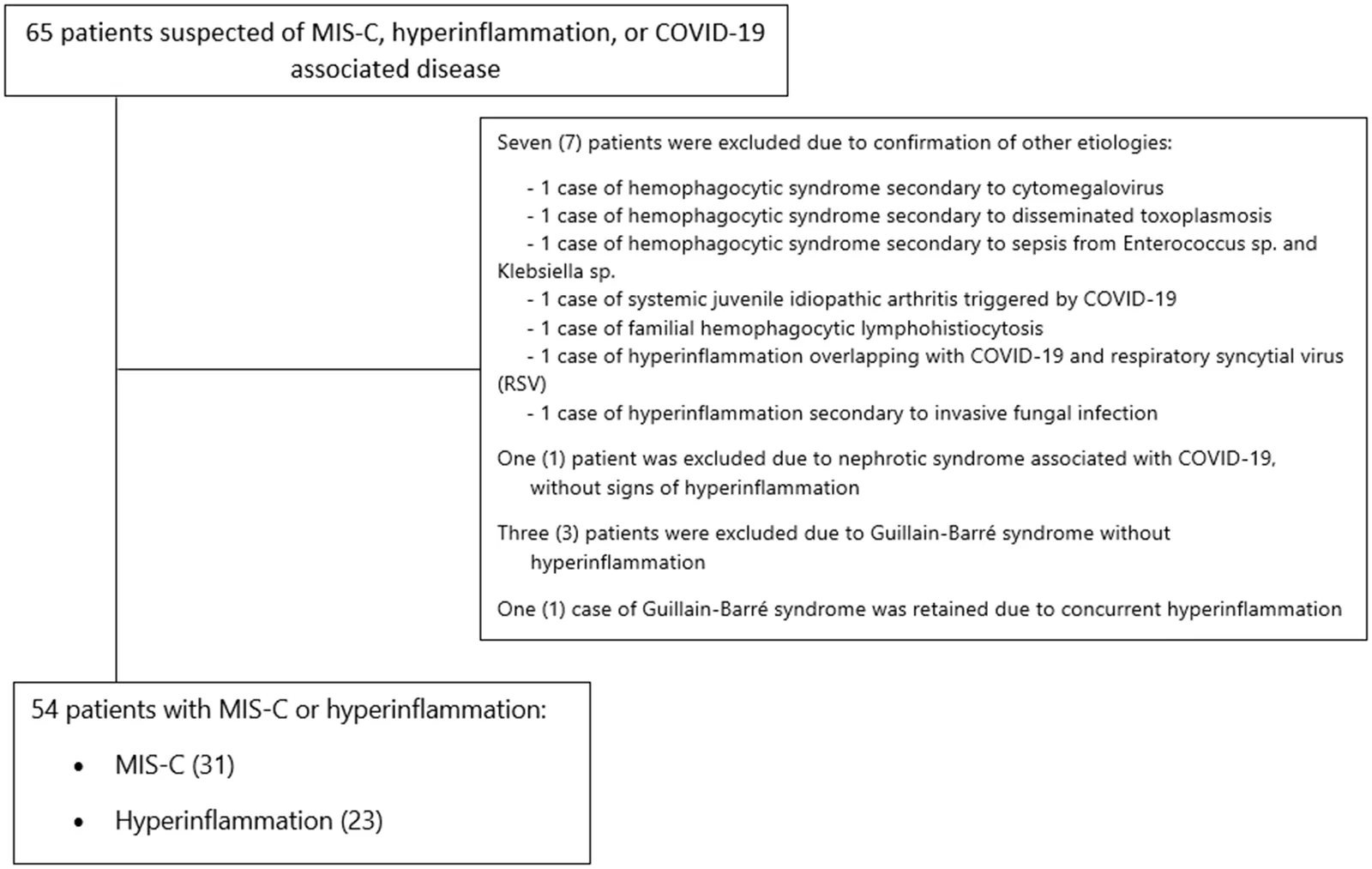
Sample description

Our sample consisted of sixty-five children and adolescents with suspected MIS-C, hyperinflammation, or conditions associated with SARS-CoV-2 infection. Among these patients, 9 did not meet the criteria for suspicion due to other confirmed etiologies accounting for the inflammatory presentation (including cytomegalovirus infection, disseminated toxoplasmosis, tuberculosis, respiratory syncytial virus, sepsis from Enterococcus *sp*. and Klebsiella *sp.*, invasive fungal infection, systemic juvenile idiopathic arthritis, and familial hemophagocytic lymphohistiocytosis) or because the duration of contact with COVID-19 was longer than 8 weeks (twice the duration considered for MIS-C suspicion). Of the sixty-five patients investigated, fifty-four were confirmed to have MIS-C (31 patients) or hyperinflammation (23 patients); 4 presented with symmetric peripheral neuropathy (Guillain-Barré Syndrome), with one case overlapping with hyperinflammation (Fig. 1).

Patients’ median age was 74 months (IQR 22.5 to 116.8). Male patients accounted for 50% (27) of the cohort. Among the studied patients, 44% (24) were of normal nutritional status, 11% (6) were obese, and 7% (4) were severely obese. In the MIS-C group, the median age was 77 months (IQR 23 to 112.5), whereas in the hyperinflammation group, the median age was 72 months (IQR 18 to 131.5). No statistically significant differences were observed between the groups regarding age, sex, ethnicity, nutritional status, or known COVID-19 contact (Table 1).

Regarding exposure to known COVID-19 contacts, 56% (30) did not have identified contacts, while only 39% (21) could identify contacts, with 28% (15) being the patients’ own parents (Table 1).

In our sample, 67% (36) of patients had some form of comorbidity, with no statistically significant difference between the groups. The most frequent comorbidity was obesity, found in 19% (10) of patients. Among patients with hyperinflammation, the second most common comorbidity was cancer, affecting 26% (6 patients in this group), while in the MIS-C group, asthma was the second most common comorbidity, affecting 23% (7 patients in this group). Other comorbidities are detailed in Table 1.

**Table 1:** Demographic, anthropometric, social and comorbidity data

Variables	Sample (54 Patients)	MIS-C (31 Patients)	Hyperinflammation (23 Patients)	*p*-value
**Demographic**				
Age (months) - Median (IQR)	74 (22,5-116,8)	77 (23–112,5)	72 (18–131,5)	> 0.9
Sex - nº (%)				0.4
- Female	27 (50)	17 (55)	10 (43)	
- Male	27 (50)	14 (45)	13 (57)	
Race/Ethnicity in the birth certificate – Number (%)				0.2
- White	43 (80)	26 (84)	17 (74)	
- Brown	2 (4)	2 (7)	0	
- Black	9 (17)	3 (10)	6 (26)	
**Anthropometric**				
Nutritional Status - nº (%)				> 0.9
- Underweight	1 (2)	1 (3,2)	-	
- Eutrophic	24 (44)	15 (48,4)	9 (39)	
- Overweight risk	3 (6)	2 (7)	1 (4)	
- Overweight	1 (2)	-	1 (4)	
- Obese	6 (11)	4 (12,9)	2 (9)	
- Severely Obese	4 (7)	2 (7)	2 (9)	
**Social**				
Positive COVID contact – nº (%)				0.2
- No	30 (56)	14 (45)	16 (70)	
- Yes				
Parents	15 (28)	10 (32)	5 (22)	
Doctors	2 (4)	2 (7)	-	
Grandparents	2 (4)	2 (7)	-	
Brother/sister	1 (2)	-	1 (4)	
- Not informed	3 (6)	2 (7)	1 (4)	
**Comorbidity -** nº (%)				0.3
- Yes	36 (67)	19 (61)	17 (74)	
Obesity	10 (19)	5 (16)	5 (22)	
* Oncologic disease	8 (15)	2 (6)	6 (26)	
Asthma	8 (15)	7 (23)	1 (4)	
Previous COVID	4 (7)	4 (13)	-	
** Other				

Nº Number of patients, IQR interquartile range, % relative to the total population described

* 5 patients with acute lymphoblastic leukemia; 1 patient with acute myeloid leukemia; 1 patient with anaplastic lymphoma; 1 patient with neuroblastoma

** 2 patients with sickle cell anemia; 2 with autism spectrum disorder; 2 with epilepsy; 2 with HIV exposure; 2 with congenital syphilis; 2 with cardiac morphological alterations (both with atrial septal defect, one with concentric left ventricular hypertrophy); 2 with hypertension; 2 with diabetes insipidus; 2 with panhypopituitarism; 2 with dysphagia; 1 with previous severe aplastic anemia; 1 with a mother positive for COVID during pregnancy; 1 with cognitive deficit; 1 with chronic granulomatous disease; 1 with chronic pericarditis; 1 with previous toxic shock; 1 with Glycogen storage disease type Ib; 1 with Turner syndrome; 1 with Pierre-Robin syndrome; 1 with congenital diaphragmatic hernia; 1 with mastocytosis; 1 with tracheomalacia; 1 with nephronophthisis; 1 with previous pulmonary and meningeal tuberculosis under treatment; 1 with adrenal insufficiency; 1 with a history of bone marrow transplantation (BMT)

### Clinical aspects

In our sample, 69% (37) of patients had a positive PCR-RT test, and 43% (23) had positive COVID serology. 100% (23) of the patients with hyperinflammation had a positive COVID RT-PCR, while it was positive in 45% (14) of the MIS-C group. Regarding serology, 68% (21) of the MIS-C group had a positive result, with 81% of these (17) showing only positive IgG (indicating previous COVID infection (Table 2).

Regarding clinical findings, the most frequent sign was fever present in 96% (52) of all patients. The frequency of symptoms varied according to the group analyzed, as detailed in Table 2. Altered mental status was observed in 61% (19 patients) of the MIS-C group and in 26% (6 patients - *p* = 0.014) of the hyperinflammation group. Cardiological symptoms were present in 48% (15 patients - *p* = 0.018) of the MIS-C group, and conjunctival hyperemia was noted in 29% (9 individuals - *p* = 0.032) of this group.

Aneurysms and serosal inflammation (serositis) were exclusive to patients with multisystem inflammatory syndrome. Arthritis and arthralgia were predominant in the MIS-C group but did not show statistical significance. In contrast, symmetric peripheral neuropathy (Guillain-Barré) was observed only in one patient in the hyperinflammation group.

Among our entire sample, 40% (22 patients) required supplemental oxygen, some with the need for ventilatory support. Specifically, 13% (7) required mechanical ventilation, 15% (8) required high-flow nasal cannula, 4% (2) required non-invasive ventilation, and 20% (11) required supplemental oxygen. Additionally, 38.9% (21) required admission to a pediatric intensive care unit, and 20% (11) required vasoactive drugs. The hyperinflammation group exhibited ventilatory dysfunction in 57% (13 children - *p* < 0.001) of patients, whereas 13% (4 children) had dysfunction in the MIS-C group.

**Table 2:** Diagnostic test and clinical data

Variables	Sample (54 Patients)	MIS-C (31 Patients)	Hyperinflammation (23 Patients)	*p*-value
**Diagnostic test**				< 0.001
RT-PCR – nº (%)				
- Positive	37 (69)	14 (45)	23 (100)	
- Negative	17 (32)	17 (55)	-	
COVID Serology – nº (%)				0.14
- Positive				
IgG positive	17 (32)	17 (55)	-	
IgG and IgM positive	6 (11)	4 (13)	2 (9)	
**Clinical signs and symptoms** – nº (%)				
- Fever	52 (96)	31 (100)	21 (91)	0.2
- Abdominal pain	35 (65)	23 (74)	12 (52)	0.094
- Cough	24 (44)	11 (35)	13 (57)	0.12
- Dermatological *	29 (55)	13 (43)	16 (70)	0.057
- Diarrhea	21 (39)	15 (48)	6 (26)	0.1
- Cardiological **	19 (35)	15 (48)	4 (17)	0.018
- Rhinorrhea	18 (33)	11 (35)	7 (30)	0.7
- Odynophagia	12 (22)	4 (13)	8 (35)	0.056
- Respiratory dysfunction	17 (32)	4 (13)	13 (57)	< 0.001
- Myocarditis	3 (6)	3 (10)	-	0.3
- Vomiting	15 (28)	10 (32)	5 (22)	0.4
- Headache	10 (19)	7 (23)	3 (13)	0.5
- Serositis	4 (7)	4 (13)	-	0.13
- Conjunctival hyperemia	10 (19)	9 (29)	1 (4)	0.032
- Arthritis/arthralgia	8 (15)	7 (23)	1 (4)	0.12
- Neurological***	25 (46)	19 (61)	6 (26)	0.014
# Other	38 (70)	28 (90)	10 (43)	< 0.001

Nº Number of patients, % relative to the total population described

* Diffuse erythematous rash or oral ulcer

** Hypotension, signs of low flow or heart rate alteration

*** Altered mental status

# Peeling of extremities, presence of stridor, aneurysms, bone marrow aplasia, loss of smell/taste, appendicitis, pancreatitis, Guillain-Barré syndrome

The most frequent laboratory findings in the MIS-C group was increased ESR (*p* = 0.001), triglycerides (*p* = 0.009), and fibrinogen (*p* = 0.03), as well as elevated troponin (*p* = 0.034), D-dimer (*p* = 0.015), and BNP (*p* = 0.02), and decreased albumin (*p* = 0.02) compared to the HI group. The hyperinflammation group exhibited a higher percentage of ferritin levels exceeding 500 ng/mL (*p* = 0.046) as shown in Table 3.

The median serum BNP level was higher in the MIS-C group, with a value of 126 pg/ml (IQR 30–770), compared to 17.5 pg/ml (IQR 10-102.3) in the hyperinflammation group (*p* = 0.048). Although other tests did not show statistical significance, the median levels of troponin and D-dimers were higher in the MIS-C group (Table 4).

Although there was statistical significance regarding the presence of elevated ESR, triglycerides, fibrinogen, troponin, D-dimers, and reduced albumin in the dichotomous analysis (presence or absence of the alteration), statistical significance was not maintained when analyzing the test values in our population.

**Table 3:** Presence of laboratory abnormality

Variables	Sample 54 Patients)	MIS-C (31 Patients)	Hyperinflammation (23 Patients)	*p*-value
**Renal Function Abnormalities**	10 (19)	7 (23)	3 (14)	0.2
- Aseptic pyuria	8 (15)	7 (23)	1 (4)	0.2
**Hematological Abnormalities**				> 0.9
- Anemia	35 (65)	20 (65)	15 (65)	> 0.9
- Leukopenia	13 (24)	5 (16)	8 (35)	0.2
- Neutropenia	23 (43)	14 (45)	9 (39)	0.8
- Lymphopenia	30 (56)	19 (61)	11 (48)	0.4
- Thrombocytopenia	19 (35)	11 (35)	8 (35)	> 0.9
**Inflammatory Abnormalities**				> 0.9
- Elevated CRP	51 (94)	28 (90)	23 (100)	0.5
- Elevated ESR	34 (63)	25 (81)	9 (39)	0.001
- Elevated Ferritin	42 (78)	26 (84)	16 (70)	0.14
>500 ng/mL	35 (83*)	20 (77*)	15 (94*)	0.046
- Elevated Triglycerides	33 (61)	22 (71)	11 (48)	0.009
- Elevated LDH	37 (69)	22 (71)	15 (65)	0.3
- Elevated Fibrinogen	33 (61)	21 (68)	12 (52)	0.03
- Reduced Albumin	27 (50)	21 (68)	6 (26)	0.002
**Cardiac and Vascular enzyme Abnormalities**				0.06
- Elevated Troponin	19 (35)	13 (42)	6 (26)	0.034
- Elevated D-dimers	45 (83)	29 (94)	16 (70)	0.015
- Elevated BNP	21 (39)	17 (55)	4 (17)	0.02
**Coagulation Test Abnormalities**	19 (35)	13 (42)	6 (23)	0.1
**Hepatic Enzyme Abnormalities**	31 (57)	18 (58)	13 (56)	0.2

**Table 4:** Laboratory values

Variables Median (IQR)	Sample (54 Patients)	MIS-C (31 Patients)	Hyperinflammation (23 Patients)	*p*-value
ESR (mm/1 h)	52.0 [19.5–81.0]	62.0 [25.5–86.3]	32.0 [7.0–66.0]	0.062
Ferritin (ng/mL)	902.6 [399.7-2,244.7]	672.4 [380.4-3,109.4]	1,192.6 [632.1-2,085.3]	0.7
Triglycerides (mg/dL)	218.0 [175.0-300.0]	224.0 [176.0-289.0]	202.0 [169.0-298.5]	0.8
Albumin (g/dL)	3.1 [2.7–3.7]	3.1 [2.7–3.7]	3.2 [2.9–3.6]	0.5
D-dimers (ug/mL)	3.1 [1.7–10.3]	5.4 [2.3–14.2]	2.7 [1.4-4.0]	0.2
BNP (pg/mL)	81.6 [17.2–682.0]	126.0 [30.0-770.0]	17.5 [10.0-102.3]	0.048
Troponin (pg/mL)	10.0 [10.0-62.3]	17.7 [10.0-64.7]	10.0 [10.0-41.7]	0.7
AST (U/L)	44.0 [23.8–124.0]	42.0 [22.0–83.0]	96.0 [26.0-175.0]	0.3

**Fig. 2:**
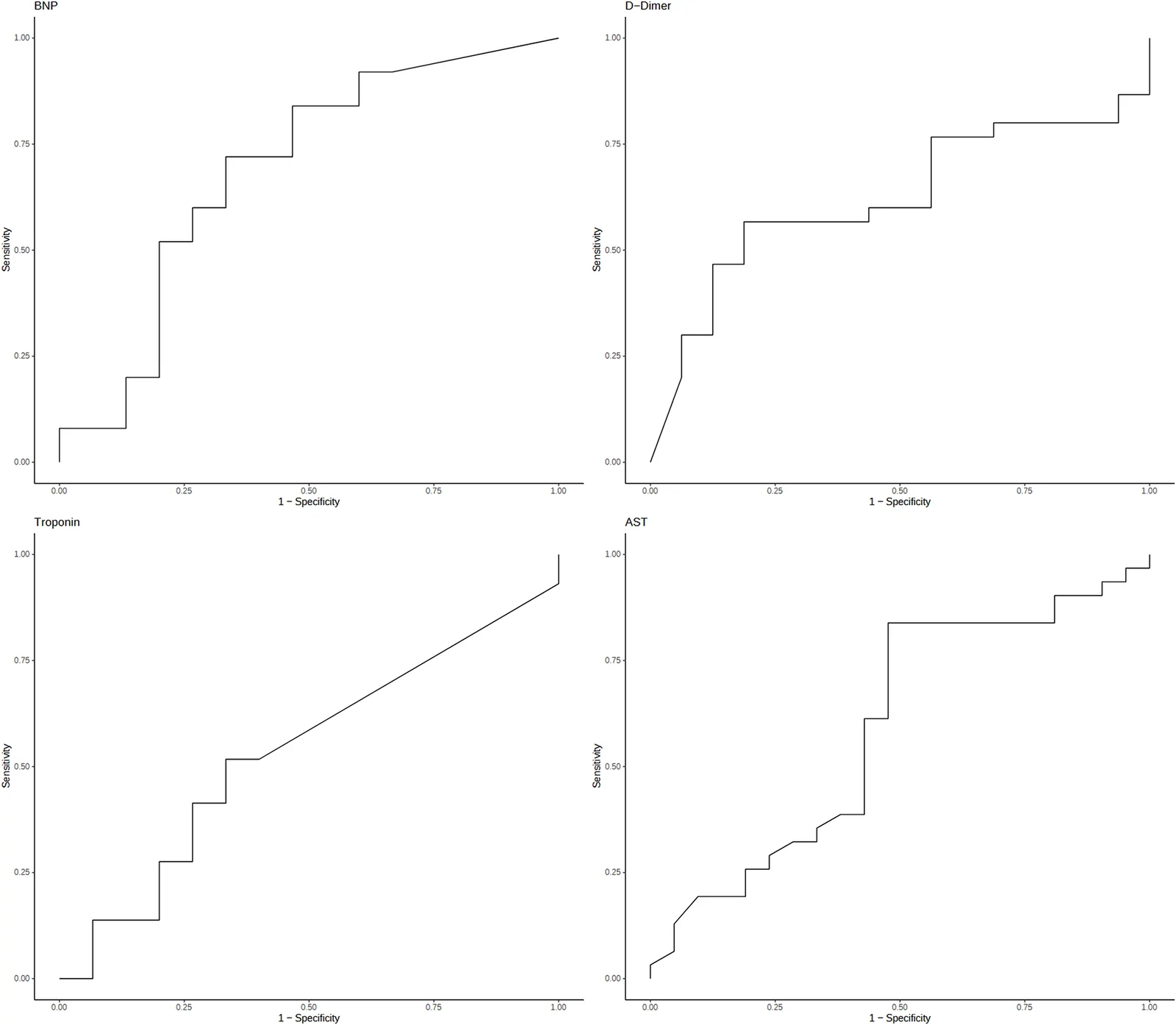
ROC curves

The ROC curves were performed for the main tests (BNP, Troponin, D-dimer, and AST) and are detailed in Table 5; Fig. 2.

**Table 5:** ROC curve values

Variable	Cut-off value	AUC	AUC CI95	Sensitivity	Specificity
BNP	52.15 (pg/mL)	0.69	0.50–0.87	72%	67%
D-dimers	4.25 (ug/mL)	0.62	0.46–0.79	67%	81%
Troponin	14.45 (pg/mL)	0.53	0.35–0.71	52%	67%
AST	93	0.59	0.43–0.76	84%	52%

For BNP measurement, a value of 52.15 pg/mL was identified as the optimal cutoff for differentiating between the groups (AUC = 0.69), with a sensitivity of 72% and specificity of 67%. Troponin testing showed a cutoff point of 14.45 pg/mL with a sensitivity of 52% and specificity of 67%, while D-dimer testing demonstrated high specificity (81%) at a cutoff of 4.25 µg/mL. The test with the best sensitivity, despite an AUC of 0.59, was AST (aspartate transaminase) with a cutoff of 93 U/L, showing a sensitivity of 84% and specificity of 52%.

The recommended treatment for inflammatory conditions associated with COVID-19 includes the use of glucocorticoids for both groups, and the use of immunoglobulin and aspirin (ASA) restricted to the MIS-C group.

Although glucocorticoids are considered the first-line treatment for both MIS-C and hyperinflammation, they were used in only 56% (30) of the sample, with 61.3% (19) of the MIS-C group and 47.8% (11) of the hyperinflammation cases receiving this treatment, with no statistically significant difference.

Regarding the use of intravenous human immunoglobulin, 74.2% (23) of the patients with MIS-C received the medication, with an average dosage of 2 g/kg. In contrast, only 17% (4) of the hyperinflammation group received immunoglobulin (all before the diagnosis of hyperinflammation), and this difference is statistically significant (*p* < 0.001). Regarding acetylsalicylic acid use, 61% (19) of the MIS-C group received this medication, while only 9% (2) of the hyperinflammation population used it due to the associated risk of thrombocytosis. This difference is also statistically significant (*p* < 0.001).

Among the patients analyzed, there were only three deaths, resulting in a mortality rate of 5.5% in the overall study population, with no statistically significant difference between groups. In our sample, there were two deaths in the hyperinflammation group among children with comorbidities (one with panhypopituitarism and another with severe asthma), while in the MIS-C group, the patient who died had primary bone marrow aplasia.

## Discussion

From this case series, it is possible to conclude that altered mental status and conjunctival hyperemia are more common in Multisystem Inflammatory Syndrome in Children (MIS-C), whereas respiratory dysfunction is more prevalent in Hyperinflammation. Regarding the more frequent laboratory findings, hypoalbuminemia, increased troponin, BNP, and d-dimers appear to be markers of MIS-C, while ferritin levels > 500 ng/mL are indicative of Hyperinflammation. However, some remarks concerning our sample are necessary.

Our overall median age was 6–9 years, similar to the population described in prior studies on the subject [17]. Unlike data from adult patients, where obesity is associated with up to a 72% increased risk of hospitalization during COVID-19 due to an increased ACE2 receptor expression [18], our population was predominantly composed of well-nourished patients and obesity was similar in both groups. Unfortunately, the lack of anthropometric data for up to 28% (15 patients) significantly hampers the nutritional assessment of these patients.

In our population, the presence of comorbidities was significant in both groups, due to our unit being a referral center for medium and high complexity pediatric care. Both groups exhibited a prevalence of comorbidities > 60%, with the most common being obesity (similarly distributed between the groups), oncological diseases, and asthma. Contrary to early assumptions during the coronavirus pandemic, asthma was not confirmed as a risk factor for severe COVID-19 [13] and was more frequent in our sample among patients with MIS-C than in those with acute disease and Hyperinflammation. The finding of oncological diseases in the Hyperinflammation group aligns with the international literature [5, 14].

Regarding the symptomatology presented, fever (an inclusion criterion for the MIS-C group) was the most common symptom in both groups. Gastrointestinal, cutaneous, and cardiological symptoms were more frequent in the MIS-C group, with the latter showing statistical significance. Respiratory and neurological symptoms were more prevalent in the Hyperinflammation group, also with statistical significance. The presence of aneurysms was exclusive to the MIS-C group, while the only case of Guillain-Barré syndrome was observed solely in the Hyperinflammation group.

Our sample recorded only three deaths, with 2 occurring in the Hyperinflammation group and 1 in the MIS-C group. When considering all patients with inflammatory complications associated with COVID-19, this results in a mortality rate of 5.5%, which is higher than that observed in Whittaker’s study in England (0.1%) [9] but lower than that reported in studies in Latin American (9%) [19]. The lower number of deaths compared to other Latin American studies may be associated with the high degree of suspicion of this condition in our hospital, the quality of care in the Pediatric Intensive Care Unit (PICU), and the support and expertise of a specialized Pediatric Rheumatology team. Additionally, the fact that deaths are predominantly concentrated in the Hyperinflammation group may be related to the difficulty in recognizing this condition, with symptoms often being diagnosed as a direct effect of SARS-CoV-2 or sepsis rather than as an immune mediated condition, thus delaying appropriate identification and treatment.

When analyzing the diagnostic and laboratory changes most frequently described in inflammatory complications of COVID-19, some aspects stand out. Erythrocyte sedimentation rate (ESR) alteration was more frequent in the MIS-C group, which was statistically significant. ESR is a marker with a delayed response to inflammation, justifying its alteration frequency in the MIS-C group, possibly associated with the time elapsed between COVID-19 infection and inflammation in this group. A significant portion of the MIS-C group exhibited elevated D-dimers, BNP, and troponin, although when analyzed, only BNP remained statistically significant (despite troponin and D-dimers showing higher interquartile ranges compared to the other group). This finding is likely due to the small sample size for assessing differences in these test values. Nonetheless, the association with altered D-dimers, BNP, and troponin is consistent with previous studies [3, 7, 17] and underscores the need for further evaluation of their diagnostic and prognostic applicability in MIS-C. D-dimer is a reliable biomarker of clot formation and subsequent degradation, thus associated with a prothrombotic state and thrombosis formation [20]. BNP is a marker of cardiac myocytes, potentially indicating either direct inflammation in cardiac cells or an indirect effect of thrombosis in the pulmonary or peripheral circulation; however, it has not yet been associated with increased mortality in COVID-19, unlike troponin levels [21]. There is a reported association between MIS-C, acute COVID-19 infection, and vascular thrombosis [22]. Regarding thromboembolic propensity, the inflammatory response associated with acute or late SARS-CoV-2 infection promotes a series of endothelial events affecting homeostasis and impairing fibrinolysis [22, 23, 24]. Our population, however, did not present any new thrombotic events during inflammatory complications from COVID-19 or during the hospitalization period.

Our study was retrospective, carrying the inherent risk that relevant signs or symptoms might not have been reported or recorded by the attending team. Dependence on pediatric emergency services and pediatric intensive care units for patient inclusion, limited interaction between pediatric rheumatology and adult care units (our hospital serves patients over 14 years of age), and the challenges in identifying MIS-C and, particularly, Hyperinflammation may have contributed to presenting a sample that is not fully representative of our reality. The Hyperinflammation group shows a lack of data for certain tests in up to 48% of the population. This lack of data may hinder comparisons between groups, sometimes driven by attributing inflammatory symptoms to acute COVID-19 and not to its inflammatory complication, as for a difficulty in following up the investigation. The number of participants and the comparison being made between two groups with inflammatory complications, rather than against a healthy population or COVID-19 cases without inflammatory complications, may have limited the statistical significance of some tests (troponin and d-dimers). In the MIS-C group, despite having larger interquartile ranges for the troponin and d-dimers alteration, the null hypothesis was not rejected. Our cut-off values in the ROC curves (specially the D-dimer value) had a similar specificity and sensitivity to MIS-C presence as the association of elevated troponin and D-dimers to myocardial involvement presented by Kostik et al. [25]. Since the study was not designed for detailed cardiac evaluation, the association of blood tests for vascular/cardiac function was not analyzed together with detailed echocardiogram findings, making it impossible to describe specific cardiac involvement and its association with cardiac function and myocardial involvement.

## Conclusions

Our study constitutes an original investigation comparing clinical and laboratory findings between Multisystem Inflammatory Syndrome in Children (MIS-C) and Hyperinflammation associated with COVID-19. Altered mental status in MIS-C and respiratory symptoms in Hyperinflammation are more frequent in each group. Additionally, regarding laboratory tests, hypoalbuminemia in MIS-C and elevated ferritin levels in Hyperinflammation are more prevalent. Further research is needed to strongly determine the cut-off values for markers such as BNP, troponin, and d-dimers for diagnosis and prognosis in the pediatric population with MIS-C but present considerable association with myocardial involvement as previously stated in the literature.

## Electronic supplementary material

Below is the link to the electronic supplementary material.

Supplementary Material 1

Supplementary Material 2

## Data Availability

Data is provided within the manuscript or supplementary information files.

## Abbreviations

ACE2: Angiotensin-converting enzyme 2
ALT: Glutamic pyruvic transaminase
AST: Glutamic oxaloacetic transaminase
BNP: Brain natriuretic peptide
CRP: C-reactive protein
ESR: Erythrocyte Sedimentation Rate
GGT: Gamma glutamyl transferase
HI: Hyperinflammation
INR: International normalized ratio of prothrombin time
LDH: Lactate Dehydrogenase
MIS-C: Multisystem Inflammatory Syndrome in Children
RT-PCR: Real-time polymerase chain reaction
