# Supplementary material for: Clinical and laboratory markers defining MIS-C and hyperinflammation in COVID-19: a cross-sectional study in a tertiary hospital
Source: Adv Rheumatol. 2025 Mar 17;65:16. doi: 10.1186/s42358-025-00447-6 (PMC13488768; doi:10.1186/s42358-025-00447-6)
Supplement: Supplementary file 1 [file 42358_2025_447_MOESM1_ESM.docx]

**Definitions:**

- **Hyperinflammation**: COVID-19-associated hyperinflammation is defined by an increase in inflammatory markers, acute SARS-CoV-2 infection (up to 4 weeks), and involvement of two or more systems or organs [5,14].
- **MIS-C**: The CDC diagnostic criteria for MIS-C used in 2022 are as described below [16]:

Clinical Criteria:

Individual under 21 years of age with:

- A minimum of 24 hours of fever (objectively or subjectively) ≥ 38°C AND
- Severe illness or hospitalization required AND
- 2 or more affected organs or systems (e.g., cardiac, renal, respiratory, hematological, gastrointestinal, dermatological, neurological)

Laboratory Evidence of Inflammation:

One or more of the following: elevated C-reactive protein, erythrocyte sedimentation rate, fibrinogen, procalcitonin, D-dimer, ferritin, LDH, or interleukin-6; elevated neutrophils or reduced lymphocytes; low albumin;

Laboratory or Epidemiological Evidence of SARS-CoV-2 Infection:

- SARS-CoV-2 positive by real-time reverse transcription polymerase chain reaction (RT-PCR), serology, or antigens OR
- Exposure to COVID-19 within the 4 weeks prior to the onset of symptoms

Exclusion of Other Diagnoses: No alternative diagnoses

- **Anthropometrics**: Regarding weight and height, children aged 0-5 years were assessed using the WHO reference tables for weight/length and weight/height according to age, which provided data on the nutritional status of the patients (underweight, normal weight, at risk of overweight, overweight, and obesity). For children older than 5 years, the WHO reference tables for Body Mass Index (BMI) were used (normal weight, overweight, obesity, and severe obesity).
- **Fever and Inflammation:** According to the CDC definition for suspected MIS-C or hyperinflammation cases, fever was defined as a temperature ≥ 38°C measured axillary, and laboratory evidence of inflammation was defined as increased levels of C-reactive protein (CRP), erythrocyte sedimentation rate (ESR), fibrinogen, D-dimer, ferritin, lactate dehydrogenase (LDH), elevated neutrophils or reduced lymphocytes, or low albumin.
- **Laboratory values:**
  - Sterile pyuria: Presence of urinary leukocytes > 10 leukocytes per field with a negative urine culture.
  - Renal function alteration: Creatinine levels exceeding 0.53 mg/dL in children <11 months, 0.55 mg/dL between 1-3 years, 0.65 mg/dL between 4-6 years, 0.69 mg/dL between 7-11 years, 0.8 mg/dL between 12-14 years, 1.1 mg/dL in boys aged 15-18 years, and 0.88 mg/dL in girls aged 15-18 years.
  - Anemia: Hemoglobin levels below 11 g/dL between 6 months-5 years, below 11.5 g/dL between 5-14 years, and below 12 g/dL in individuals over 15 years.
  - Leukopenia: Total leukocytes count below 4000/µL, and leukocytosis: Values above 14000/µL.
  - Neutropenia: Segmented counts below 1500/µL.
  - Lymphopenia: Lymphocytes count below 3000/µL in <2 years, below 2000/µL between 2-6 years, and below 1000/µL from age 7 onwards.
  - Thrombocytopenia: Platelet counts below 200,000/µL.
  - Altered CRP: Values above 5 mg/L.
  - Altered ESR: Values above 10 mm in the first hour.
  - Increased ferritin: Values above 204 ng/mL in female patients and above 274.66 ng/mL in male patients.
  - Altered LDH: Values above 220 U/L.
  - Reduced fibrinogen: Values below 200 mg/dL.
  - Increased triglycerides: In non-fasting children, values above 85 mg/dL between 2-9 years, and above 100 mg/dL between 9-19 years.
  - Reduced albumin: Values below 2.8 g/dL in <11 months, below 3.8 g/dL between 1-7 years, below 4.1 g/dL between 8-14 years, and below 4 g/dL between 15-18 years.
  - Altered troponin: Values above 50 pg/mL.
  - Altered D-dimer: Values above 0.5 µg/mL.
  - Altered BNP: Values above 100 pg/mL.
  - Coagulation test alterations: INR above 1.2 and aPTT above 36.7 seconds without prior anticoagulation use.
  - Altered AST (TGO): Values above 67 U/L in children <11 months, above 44 U/L between 1-6 years, above 36 U/L between 7-11 years, and above 35 U/L between 13-18 years.
  - Altered ALT (TGP): Values above 33 U/L in children <11 months, above 25 U/L between 1-12 years, and above 24 U/L between 13-18 years.
  - Altered GGT: Values above 127 U/L in children <11 months, and above 21 U/L between 1-18 years.
